# Transvaginal Ultrasound as a First-Line Approach in Deep Endometriosis: A Pictorial Essay

**DOI:** 10.3390/diagnostics11030444

**Published:** 2021-03-04

**Authors:** Bogdan Doroftei, Radu Maftei, Ovidiu-Dumitru Ilie, Gabriela Simionescu, Emil Anton, Theodora Armeanu, Ana-Maria Dabuleanu, Elena Mihalceanu, Constantin Condac, Ciprian Ilea

**Affiliations:** 1 Faculty of Medicine, University of Medicine and Pharmacy “Grigore T. Popa”, University Street, no 16, 700115 Iasi, Romania; bogdandoroftei@gmail.com (B.D.); gabi.ginecologie@gmail.com (G.S.); emil.anton@yahoo.com (E.A.); theodoraarmeanu@yahoo.com (T.A.); dabu_93@yahoo.com (A.-M.D.); emih2001@yahoo.com (E.M.); cilea1979@yahoo.com (C.I.); 2 Clinical Hospital of Obstetrics and Gynecology “Cuza Voda”, Cuza Voda Street, no 34, 700038 Iasi, Romania; 3 Origyn Fertility Center, Palace Street, no 3C, 700032 Iasi, Romania; 4 Department of Biology, Faculty of Biology, “Alexandru Ioan Cuza” University, Carol I Avenue, no 20A, 700505 Iasi, Romania; 5 Faculty of Medicine, “Lucian Blaga” University, Victoriei Avenue, no 10, 550024 Sibiu, Romania; costicondac@gmail.com

**Keywords:** deep endometriosis, diagnosis, ultrasound, adenomyosis

## Abstract

Endometriosis (EMS) is a benign condition characterized by a systemic inflammation that affects fertile women at reproductive age. Ultrasound became, in recent years, the method of choice for both effective diagnostic and preoperative planning. Therefore, accurate characterization and mapping of endometriotic lesions is imperative in such circumstances to enable optimal approach of treatment, whether surgical or non-surgical based on the severity of the findings. This pictorial essay outlines a practical approach to evaluating patients with deep endometriosis by means of transvaginal ultrasound. The technical aspects are in conjunction with both consensus of the International Deep Endometriosis Analysis (IDEA) group and the hands-on experience acquired through daily clinical practice.

## 1. Introduction

Endometriosis (EMS) is a chronic disease defined by the presence of endometrial-like tissue outside the uterine lining. Women at reproductive age are at higher risk because it causes physiological changes that are directly correlated with fertile status. EMS incidence was estimated to be between 5 and 10% [1,2], but a series of other aspects should be taken into consideration such as the use of combined oral contraceptives, lack of routine checks at the doctor, or even misdiagnosis. Cumulatively, all those mentioned early led to a still challenging and hard to diagnose disorder.

Retrospectively, the first paper that signaled the use of ultrasound in the diagnosis of endometriosis was published in 1978 [3]. Since then, numerous studies have focused on ultrasound findings in deep endometriosis (DE) and a consensus was reached in 2016 regarding the nomenclature, definitions and measurements of endometriotic lesions depending on their localization [4].

Ultrasound for these specific lesions is a topic that has been the promoter of many other articles published. Furthermore, the objective was to test through the prism of specificity and sensibility with other protocols involving magnetic resonance imaging (MRI) and laparoscopy. From our point of view, ultrasound should become routine for DE diagnosis. Unfortunately, it is still limited to diagnosis of ovarian endometrioma [5,6,7].

This paper aims to reiterate the importance of ultrasound in the diagnosis of DE. It should become mandatory for gynecologists to be familiar with the ultrasound aspects of DE and use this diagnostic tool in daily practice following the steps proposed in the IDEA (International Deep Endometriosis Analysis group) consensus statement.

## 2. Materials and Methods

The transvaginal ultrasound technique used for detecting DE was performed according to IDEA guidelines [4].

### 2.1. Study Participants

A total of twenty-one women (mean 33.6, range 25–43 years) participated, each one reporting painful symptoms associated with endometriosis for at least 12 months (dysmenorrhea, dyspareunia or dysuria).

### 2.2. Protocol

Prior to the actual procedure, each woman underwent an MRI investigation. Endometriotic lesions were subsequently confirmed following a histologic examination and surgery.

All patients suspected were kindly advised to follow a low residue diet between 2 and 3 days and to take oral laxatives a few hours before the intervention. We further recommend patients partially empty their bladder for better visualization of endometriotic lesions.

The images with endometriosis-like symptoms were obtained from patients by using a Voluson E8 expert ultrasound machine (GE Healthcare, Chicago, IL, USA) at the Origyn Fertility Center, Iasi, Romania.

### 2.3. Procedure

At the beginning of the ultrasound examination, the uterus and adnexa were evaluated to identify and describe separately every endometriotic lesion. The presence of “soft markers” was assessed by bimanual examination: a transvaginal probe was gently pushed towards the ovary and transabdominal pressure was applied with the other to notice if the ovary would glide freely along the pelvic sidewall and uterus. The status of the Douglas pouch was appraised using the “sliding-sign” technique. If the rectum glided freely against the uterus and the posterior vaginal fornix, this would be considered as a positive “sliding sign”. The examination concluded with a survey of deep endometriosis in the anterior and posterior compartments.

### 2.4. Follow-Up

Patients were under continuous postoperative follow-up for at least 1 year to evaluate the evolution of symptoms. It should be also mentioned that were placed under standard treatment for at least 3 months on postoperative hormones (dienogest or combined oral contraceptive (COC) in continuous administration between 90–120 days, respectively).

### 2.5. Ethical Approval

The study was approved by the Ethical Committee of the Origyn Fertility Center (no 117/565/January/25/2021). Additionally, all patients gave their signed consent for ultrasound images presented in this paper to be used for research and publication purposes.

## 3. Results and Discussions

### 3.1. ADNEXA

The assessment of the adnexa involves the evaluation of ovaries, fallopian tubes and the detection of “soft markers”. A clear mapping of the left and right side should be performed especially in the cases of patients with ovaries kissing and important anatomic distortion; also, the examiner must try to establish the origin of the lesion, weather ovary, uterus, fallopian tube or other organs. For the description of ovarian lesions, we used the terminology proposed by the International Ovarian Tumor Analysis (IOTA) group [8].

**Figure 1:**
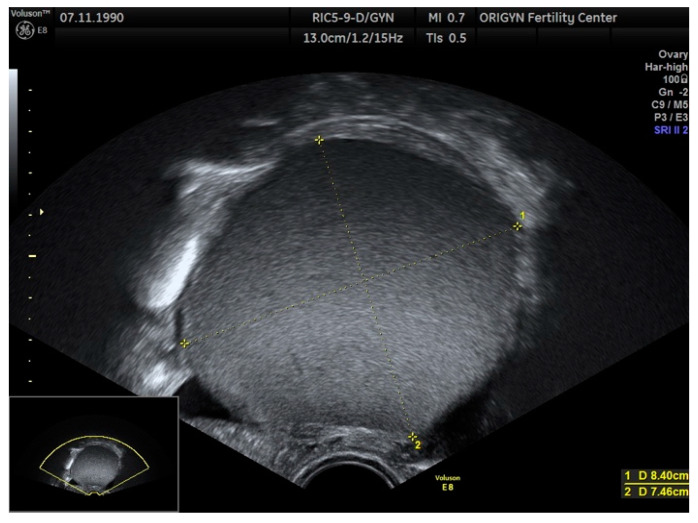
Typical aspect of an endometrioma. Unilocular cyst with “ground-glass” echogenicity.

Endometrioma or ovarian endometriosis is one of the most common forms of endometriosis with characteristic aspects of a cystic lesion with “ground-glass” echogenicity (Figure 1) and low Doppler signal. 

**Figure 2:**
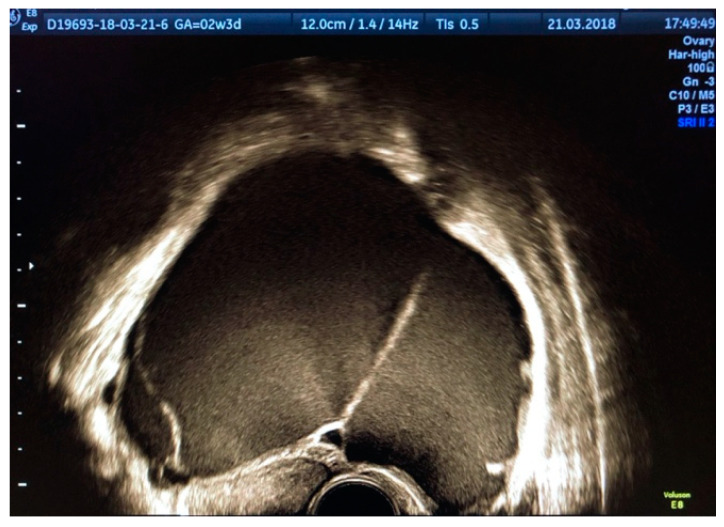
Multilocular endometrioma with “ground-glass” appearance.

**Figure 3:**
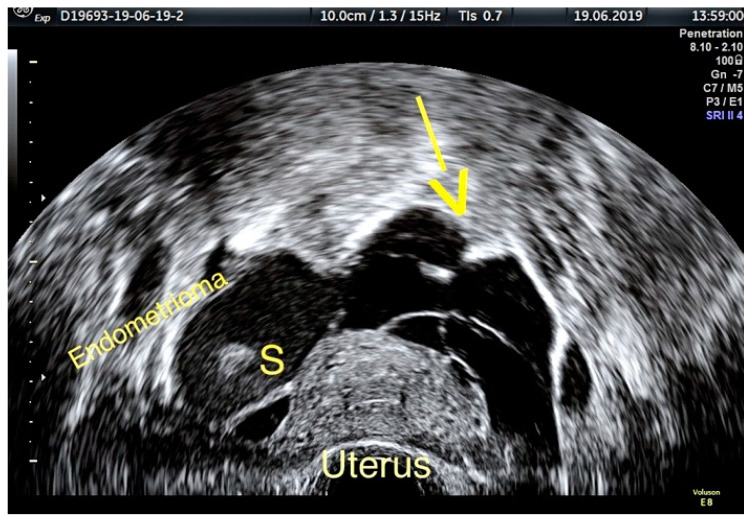
Ovarian endometrioma containing a hyperechoic area (S). Paraovarian multiple adhesions and free fluid (yellow arrow).

**Figure 4:**
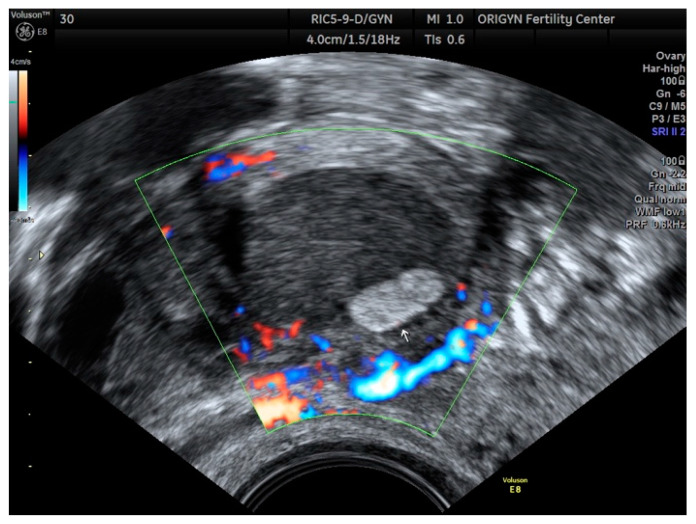
Ovarian endometrioma containing a hyperechoic area (white arrow) without Doppler signal.

According to a recent study, the ultrasound appearance of endometrioma changes with the patient’s age [9]. While in premenopausal women 75% of lesions have a “ground-glass” echogenicity, only 62% of lesions in peri- and post-menopausal women have this aspect. In addition to the typical appearance of unilocular cysts with “ground-glass” echogenicity, atypical forms of endometrioma can also be found, such as multilocular cysts (Figure 2), papillary projections or solid areas (Figure 3 and Figure 4).

**Figure 5:**
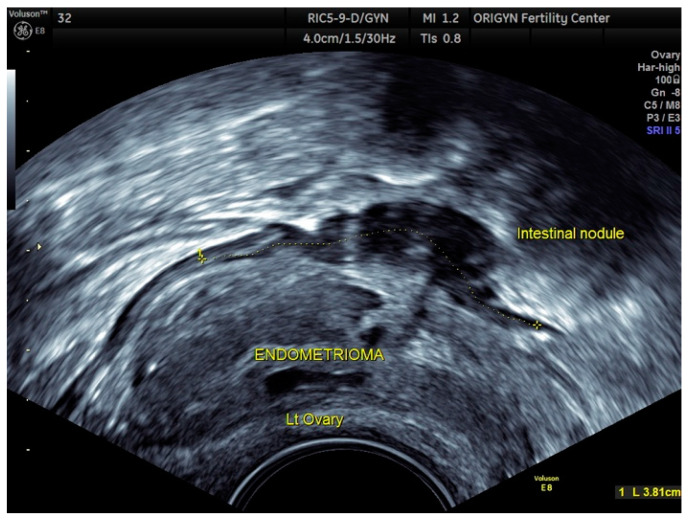
Left ovary with an endometrioma having a typical appearance. Right behind the ovary, a loop of the small intestine with an endometriotic nodule is very adherent to the ovary.

In the presence of endometrioma, due to adhesions, the fallopian tubes can be affected, resulting in hydrosalpinx or hematosalpinx. In one study, ovarian endometrioma was linked to the severity of deep endometriosis, thus having the potential to serve as a marker and a warning sign of gynecologists [10]. In addition, the surrounding ovarian bowel should be carefully checked for the presence of endometriotic lesions (Figure 5).

The evaluation of soft markers for endometriosis detected by transvaginal ultrasound includes ovarian mobility and site-specific tenderness (SST). The assessment of ovarian mobility is made by applying light pressure with the vaginal transducer in the direction of the ovary. In some cases, the operator can use his/her left hand to apply pressure on the iliac fossa region for a better evaluation of ovarian mobility. Normally, the ovary should glide freely along the pelvic sidewall and uterus. The assessment of the SST consists of applying pressure with the probe and asking the patient about the onset and site of pain. We recommend that the assessment of SST be done at the end of the examination to reduce the patient’s physical and psychological discomfort.

### 3.2. Adenomyosis

The uterine evaluation should be done according to the Morphological Uterus Sonographic Assessment (MUSA) consensus for a better characterization of lesions, especially when in the case of associated pathology like myoma [11]. Adenomyosis is a benign condition defined by the presence of endometrial glands and stroma at the level of the uterine muscle wall. The existence of endometrial-like tissue in the uterine wall induces hypertrophy and hyperplasia of the surrounding myometrium, creating a globose aspect of the uterus and an increased uterine volume.

**Figure 6:**
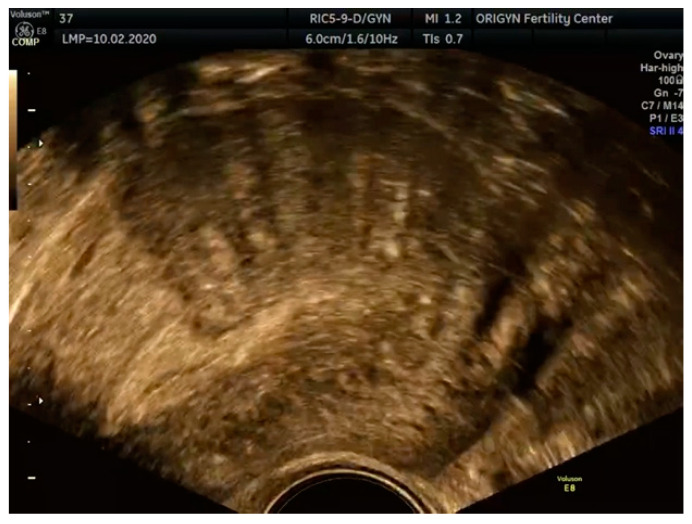
Ultrasound image of diffuse adenomyosis showing an enlarged uterus with fan-shaped shadowing: enlarged uterus with loss of the normal aspect of the myometrium: multiple myometrial cysts, fan-shaped shadowing, echogenic islands and an interrupted junctional zone.

**Figure 7:**
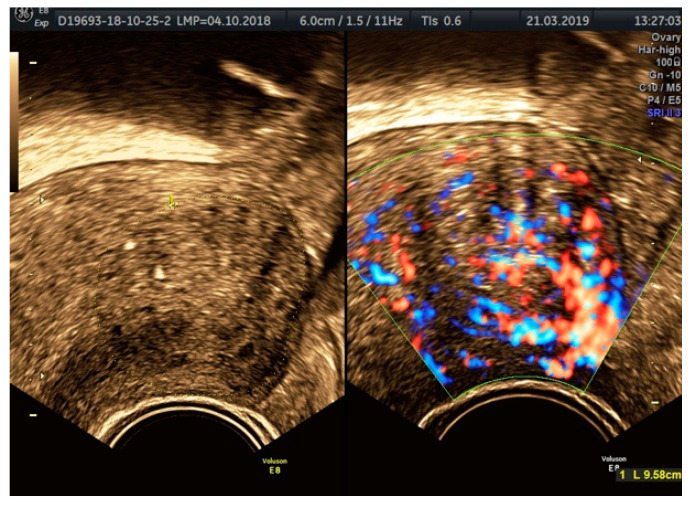
Ultrasound image of diffuse adenomyosis showing translesional blood flow in Doppler mode.

**Figure 8:**
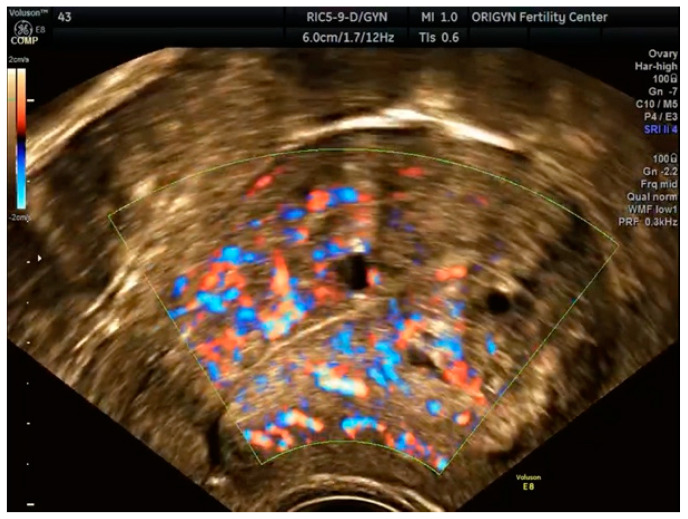
Ultrasound aspects of focal adenomyosis: myometrial cysts surrounded by echogenic rim and blood flow in Doppler mode.

**Figure 9:**
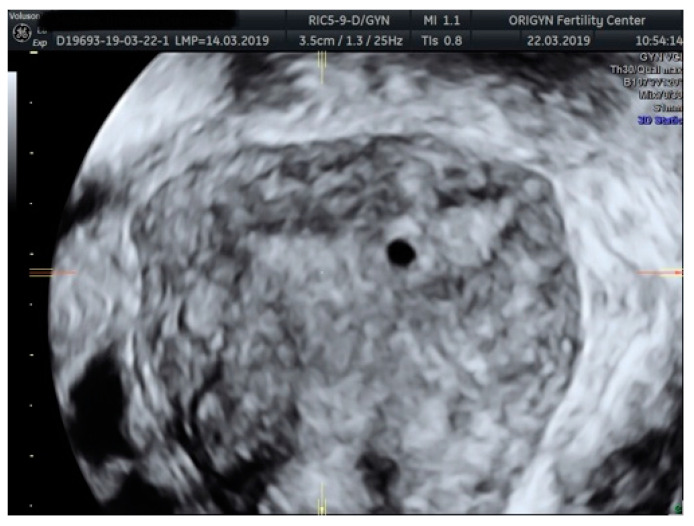
Focal adenomyosis in 3D coronal view showing cystic lesions.

Based on the histologic and ultrasonographic aspects, we can distinguish three types of adenomyosis: diffuse adenomyosis (Figure 6 and Figure 7), focal adenomyosis (Figure 8 and Figure 9) and adenomyoma.

### 3.3. Posterior Compartment

The last step of the IDEA approach consists of assessing the posterior compartment, which includes: the uterosacral ligaments (USLs), the posterior vaginal fornix, the rectovaginal septum and the bowel. We recommend starting this evaluation with the bowel because 9–22% of all women with proven endometriosis will have this type of lesions [12]. With the probe oriented in the direction of the sacrum bone, the pressure is applied gently on the entrance into the vagina. Once the bowel wall is identified, slow progression is made following the bowel curves. Most commonly, the lesions are located in the anterior wall of the bowel, but sometimes it may be possible to find an endometriotic nodule in the lateral wall or even in the posterior wall of the bowel. This is the reason why we suggest assessing the bowel in the longitudinal and transversal planes.

**Figure 10:**
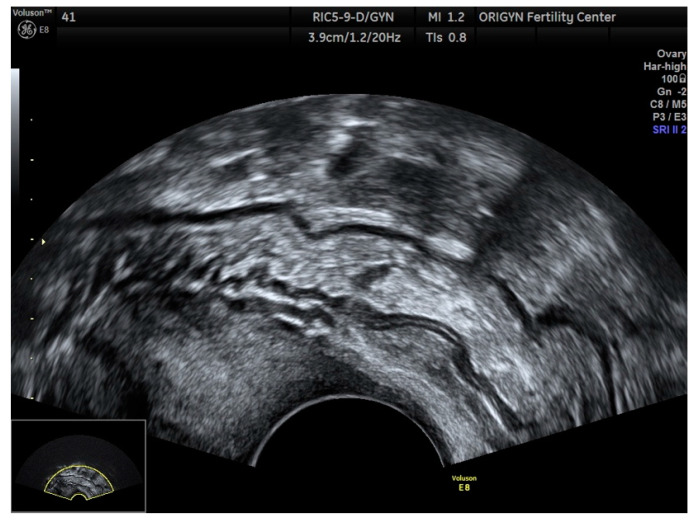
Ultrasound appearance of a normal bowel loop.

The normal thickness of the rectosigmoid wall is 1–2 mm (Figure 10). 

**Figure 11:**
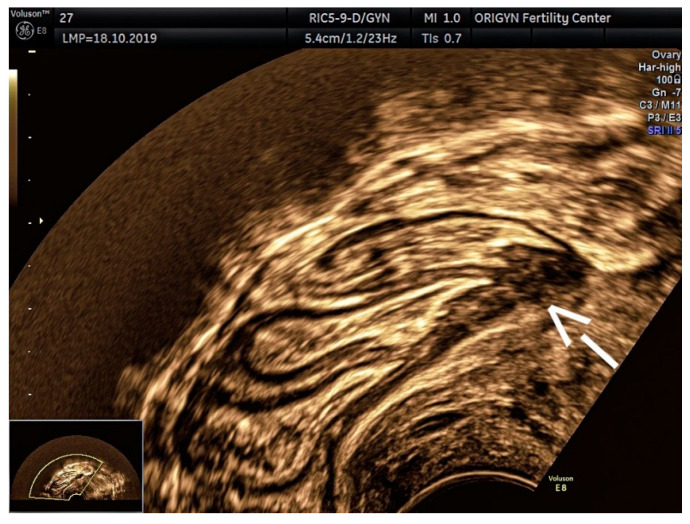
Ultrasound image showing the bowel with an endometriotic nodule (white arrow, “pulling sleeve sign”) adherent to the uterine torus, in extrinsic retraction. In this case the sliding sign was absent.

**Figure 12:**
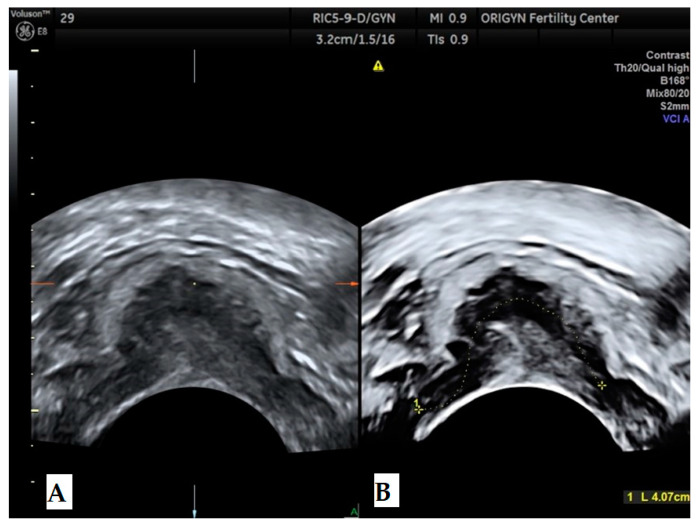
(**A**) Ultrasound image showing a deep infiltrating endometriosis (DIE) nodule with a regular outline (absence of “spikes”). (**B**) the same nodule is viewed in 3D with TUI (tomographic ultrasound imaging).

**Figure 13:**
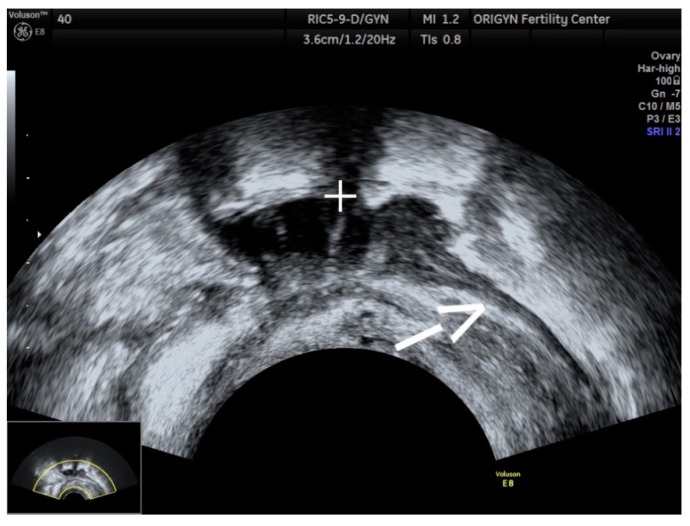
Ultrasound image showing a DIE nodule (+) with progressive narrowing, like a “tail” (white arrow), also known as a “comet” sign.

**Figure 14:**
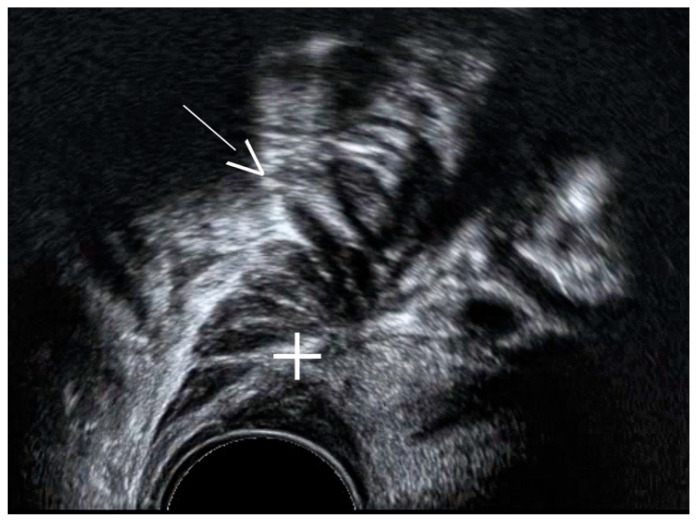
Ultrasound image showing a DIE nodule (+) with prominent spikes towards the bowel lumen (white arrow, “Indian headdress sign”).

**Figure 15:**
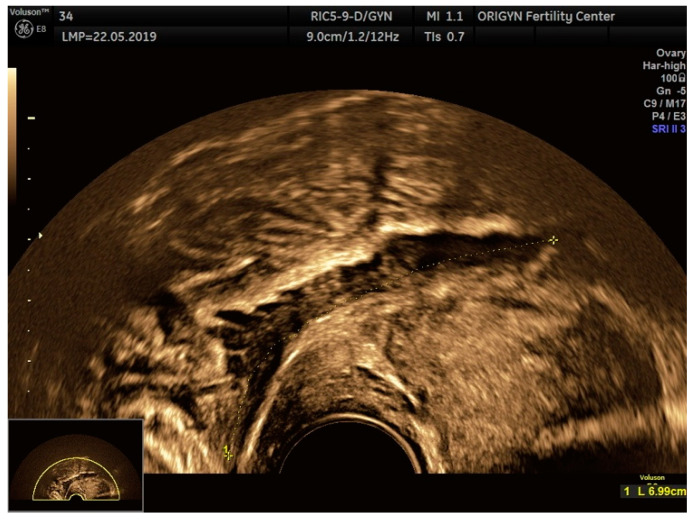
Ultrasound image showing a big DIE nodule with some spikes towards the bowel lumen, in extrinsic retraction. In this case, the sliding sign was absent.

Any focal thickening of the bowel wall that suggests an endometriotic nodule should be followed by gentle pressure with the probe to elongate the intestinal loop and to exclude an artifact or superposition of images. Another helpful tip is to observe the peristalsis of the surrounding bowel because the nodule will remain immobile. Any lesions detected during the ultrasound examination should be described under the IDEA consensus statement (Figure 11, Figure 12, Figure 13, Figure 14 and Figure 15).

**Figure 16:**
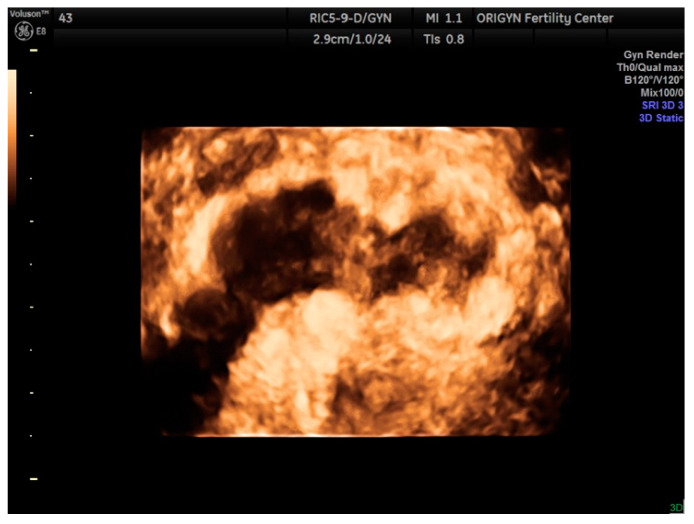
Three-dimensional findings of a rectosigmoid nodule in the coronal plane.

When evaluating intestinal endometriotic nodules, the 3D ultrasound has demonstrated its usefulness, because such imagery allows for better visualization of lesion borders (Figure 16).

**Figure 17:**
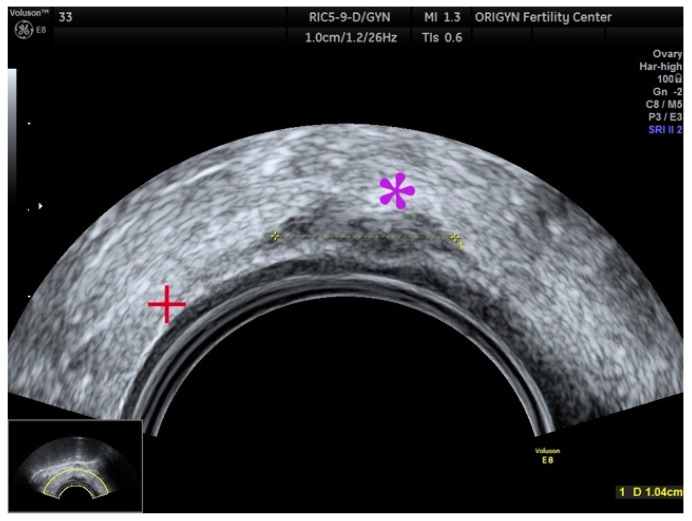
Ultrasound image depicting an endometriotic nodule of the RVS. (+) the vaginal wall as a hypoechogenic line and an endometriotic nodule (*) with discreet vaginal wall infiltration.

Another important region that needs to be checked for DE is the rectovaginal septum (RVS). This is located in the retroperitoneum, between the posterior wall of the vagina and the anterior wall of the rectum. This structure establishes a strong connection between the vagina and the rectum, containing collagen, elastic fibers, small vessels, smooth muscle cells and nerves from the inferior hypogastric plexus. The ultrasound examination of the RVS is made with the probe oriented in the same manner as for bowel evaluation. According to the IDEA group definition, the nodule should be visualized below the line passing along the lower border of the posterior lip of the cervix (Figure 17) [4]. 

It is very important to use this landmark for better differentiation between RVS endometriosis and retrocervical endometriosis. In the former case, the anterior wall of the rectum is usually infiltrated, which implies bowel resection during surgery, while in the case of retrocervical endometriosis, the bowel wall is not affected, so a local excision or ablation of the lesion is sufficient.

**Figure 18:**
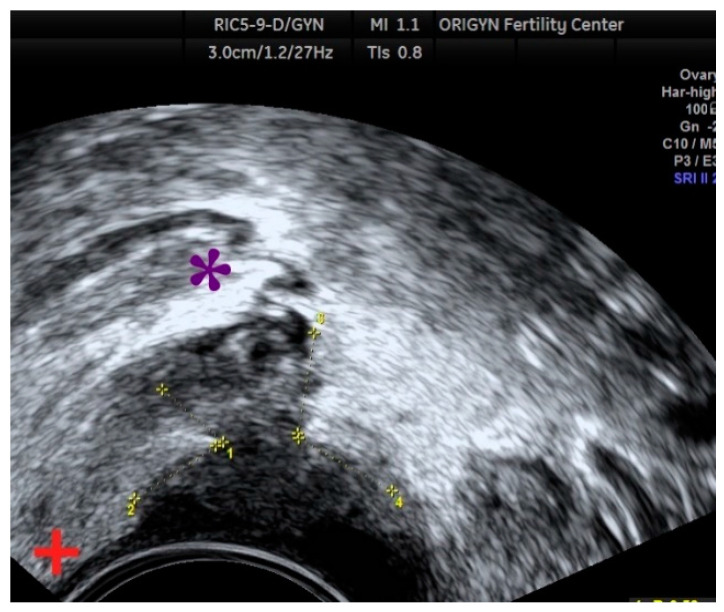
Ultrasound image showing a big nodule infiltrating the posterior vaginal fornix and the anterior wall of the rectum. (+) the vaginal wall, (*) the bowel. Diablo-like nodule.

The ultrasound evaluation of the posterior fornix should be a tenderness-guided examination. The ultrasound beam is gently placed into the posterior fornix and the patient is asked to inform the examiner about the onset and the site of any pain experienced during the examination. In addition to the size of the lesion, the operator should pay attention to the relationship with other endometriotic lesions and evaluate the mobility and the status of the sliding sign (Figure 18).

**Figure 19:**
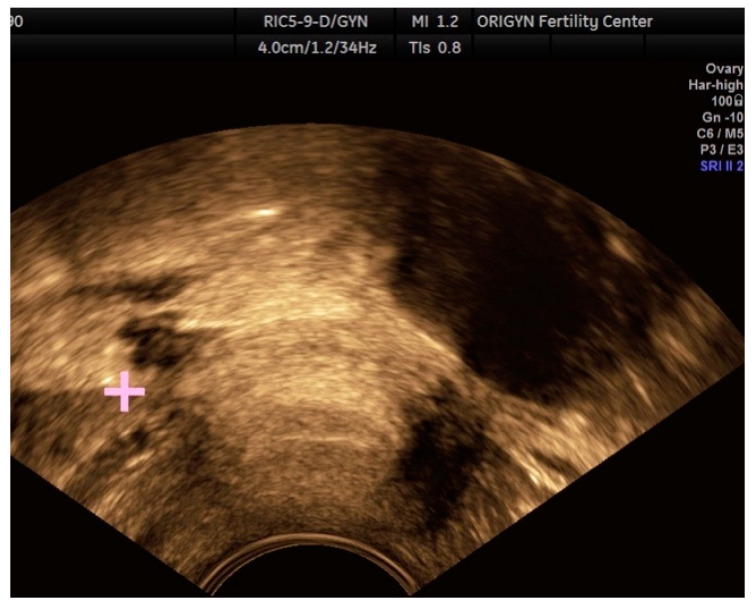
Transverse image of the cervix with a uterosacral ligaments (USL) DE hypoechoic nodule (+), with irregular outline.

**Figure 20:**
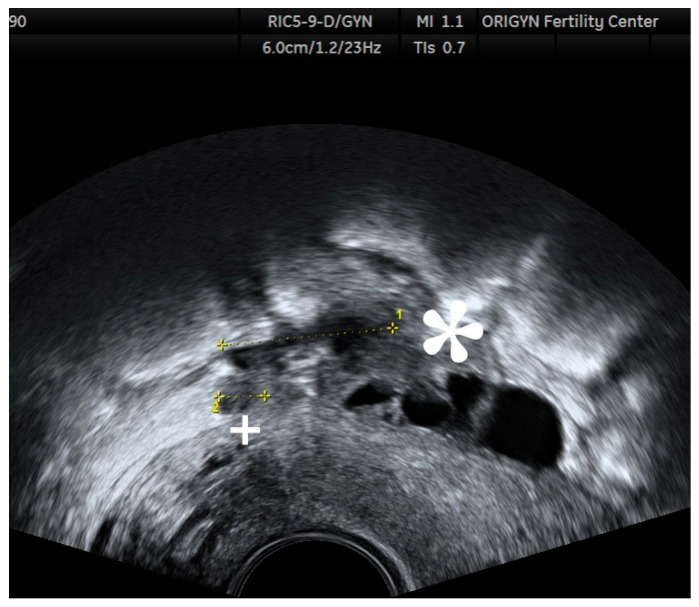
Sagittal image of the cervix showing a hypoechoic USL DE nodule (+) along with a bowel nodule adherent to a DE nodule of the uterine torus and a USL nodule.

Normally, uterosacral ligaments are two anatomic structures invisible to ultrasound examination and, according to one meta-analysis [13], the overall pooled sensitivity of transvaginal sonography (TVS) for detection of USL endometriosis was only 53% (95% confidence interval (CI), 35–70%). During the examination, the ultrasound beam is gently placed into the posterior fornix, at the midline, in a sagittal plane and then the probe is swept inferolateral to the cervix. Endometriotic lesions of the USL appear as a hypoechoic thickening and may be isolated or may be included in a big DE nodule (Figure 19 and Figure 20). The nodule should be measured in three orthogonal planes.

**Figure 21:**
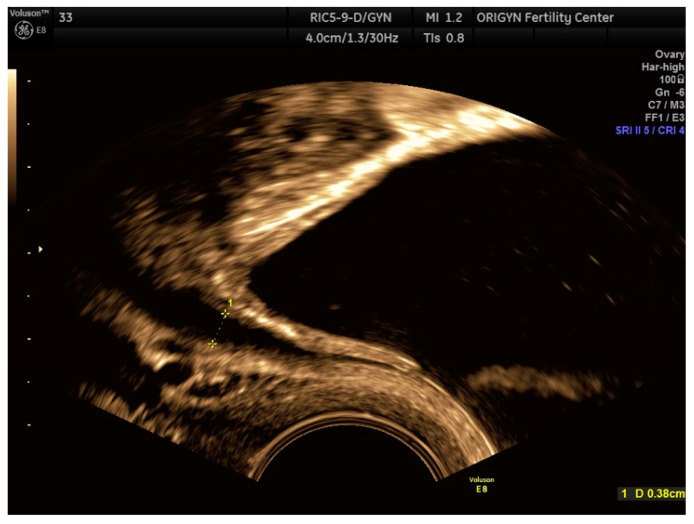
Transvaginal ultrasound of a normal ureter during peristalsis. Notice the flow of urine into the bladder.

Another important step in the evaluation of the ureter. Ureteral endometriosis may be intrinsic or extrinsic, affecting one or both ureters. Intrinsic ureteral endometriosis is due to direct infiltration of the ureteral wall by the endometrial glands and stroma. Extrinsic endometriosis is more common and is a consequence of an intensive fibrotic process due to DE. The pelvic portion of the ureters can easily be evaluated by transvaginal ultrasound. The first step is to identify the urethra in a longitudinal section and then the probe should be moved slowly to each part of the lateral pelvic wall. Ureters appear as hypoechoic tubular structures surrounded by a hyperechoic layer, measuring around 1.7 mm at rest and 2.9 mm during peristalsis (Figure 21). 

**Figure 22:**
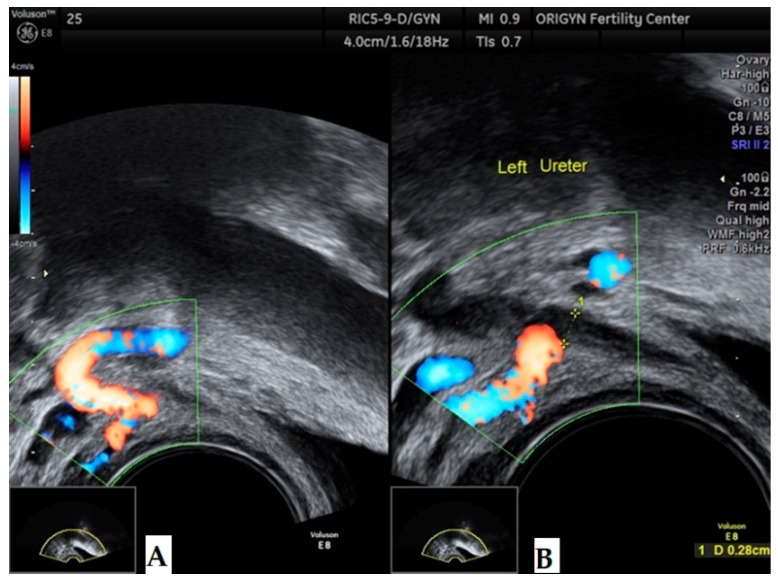
Transvaginal ultrasound of a normal left ureter (**A**) and of the crossing of the uterine artery when viewed in Doppler mode (**B**).

**Figure 23:**
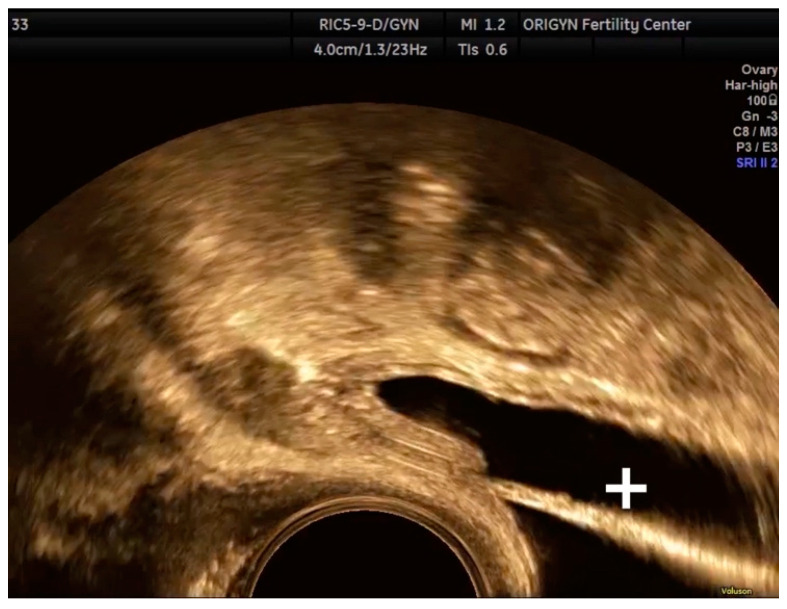
Transvaginal ultrasound of the bladder showing the ureter and the JJ stent in a normal position. (+ sign in the figure plays the role of a pointer to highlight a specific feature).

As in the case of bowel lesions, if a ureteral nodule is visualized when the probe is fixed, the nodule will remain immobile during peristalsis. Usually, the ureters are narrowed by DE at the level of the crossing with the uterine artery. This is the reason why we recommend the inspection of the ureter until this level with the ultrasound device in Doppler mode (Figure 22). In the case of a urethral JJ stent, TVS is very helpful in evaluating the position and peristalsis (Figure 23).

## 4. Conclusions

Endometriosis is a chronic disease taking a heavy toll on the quality of life and the fertility potential of the women affected by it. Considerable diagnostic delays of up to 8 years from the onset of symptoms add a further economic and social burden [14,15,16]. Therefore, we should strive towards early and comprehensive diagnosis using all means available, such as by promoting and conducting transvaginal ultrasound examinations on a larger scale. Although the transvaginal ultrasound is known to be an easy, safe and accurate method to diagnose DE, it is yet to become a routine and widely used first-line imagistic approach.

A detailed ultrasound examination using the steps of the IDEA consensus statement could detect endometriotic lesions that would otherwise be omitted during laparoscopy (i.e., bowel lesions, ureteral nodules) and contribute to a correct referral of patients suffering from advanced endometriosis to centers of excellence in endometriosis, where they can undergo minimally invasive surgery.

## Data Availability

The datasets used and analyzed during the current study are available from the corresponding author on reasonable request.

## References

[1] Vercellini P., Viganò P., Somigliana E., Fedele L. (2014). Endometriosis: Pathogenesis and treatment. Nat. Rev. Endocrinol..

[2] Viganò P., Parazzini F., Somigliana E., Vercellini P. (2004). Endometriosis: Epidemiology and aetiological factors. Best Pract. Res. Clin. Obstet. Gynaecol..

[3] Sandler M.A., Karo J.J. (1978). The Spectrum of Ultrasonic Findings in Endometriosis. Radiology.

[4] Guerriero S., Condous G., van den Bosch T., Valentin L., Leone F.P.G., Van Schoubroeck D., Exacoustos C., Installé A.J.F., Martins W.P., Abrao M.S. (2016). Systematic approach to sonographic evaluation of the pelvis in women with suspected endometriosis, including terms, definitions and measurements: A consensus opinion from the International Deep Endometriosis Analysis (IDEA) group. Ultrasound Obstet. Gynecol..

[5] Bazot M., Daraï E. (2005). Sonography and MR imaging for the assessment of deep pelvic endometriosis. J. Minim. Invasive Gynecol..

[6] Hudelist G., English J., Thomas A.E., Tinelli A., Singer C.F., Keckstein J. (2011). Diagnostic accuracy of transvaginal ultrasound for non-invasive diagnosis of bowel endometriosis: Systematic review and meta-analysis. Ultrasound Obstet. Gynecol..

[7] Guerriero S., Ajossa S., Orozco R., Perniciano M., Jurado M., Melis G.B., Alcazar J.L. (2016). Accuracy of transvaginal ultrasound for diagnosis of deep endometriosis in the rectosigmoid: Systematic review and meta-analysis. Ultrasound Obstet. Gynecol..

[8] Timmerman D., Valentin L., Bourne T.H., Collins W.P., Verrelst H., Vergote I. (2000). Terms, definitions and measurements to describe the sonographic features of adnexal tumors: A consensus opinion from the International Ovarian Tumor Analysis (IOTA) group. Ultrasound Obstet. Gynecol..

[9] Guerriero S., Van Calster B., Somigliana E., Ajossa S., Froyman W., De Cock B., Coosemans A., Fischerová D., Van Holsbeke C., Alcazar J.L. (2016). Age-related differences in the sonographic characteristics of endometriomas. Hum. Reprod..

[10] Chapron C., Pietin-Vialle C., Borghese B., Davy C., Foulot H., Chopin N. (2009). Associated ovarian endometrioma is a marker for greater severity of deeply infiltrating endometriosis. Fertil. Steril..

[11] Van den Bosch T., Dueholm M., Leone F.P.G., Valentin L., Rasmussen C.K., Votino A., Van Schoubroeck D., Landolfo C., Installé A.J.F., Guerriero S. (2015). Terms, definitions and measurements to describe sonographic features of myometrium and uterine masses: A consensus opinion from the Morphological Uterus Sonographic Assessment (MUSA) group. Ultrasound Obstet. Gynecol..

[12] Chapron C., Chopin N., Borghese B., Foulot H., Dousset B., Vacher-Lavenu M.C., Vieira M., Hasan W., Bricou A. (2006). Deeply infiltrating endometriosis: Pathogenetic implications of the anatomical distribution. Hum. Reprod..

[13] Guerriero S., Ajossa S., Minguez J.A., Jurado M., Mais V., Melis G.B., Alcazar J.L. (2015). Accuracy of transvaginal ultrasound for diagnosis of deep endometriosis in uterosacral ligaments, rectovaginal septum, vagina and bladder: Systematic review and meta-analysis. Ultrasound Obstet. Gynecol..

[14] Dunselman G.A.J., Vermeulen N., Becker C., Calhaz-Jorge C., D’Hooghe T., De Bie B., Heikinheimo O., Horne A.W., Kiesel L., Nap A. (2014). ESHRE guideline: Management of women with endometriosis †. Hum. Reprod..

[15] Nisenblat V., Bossuyt P.M.M., Farquhar C., Johnson N., Hull M.L. (2016). Imaging modalities for the non-invasive diagnosis of endometriosis. Cochrane Database Syst. Rev..

[16] Nisenblat V., Prentice L., Bossuyt P.M.M., Farquhar C., Hull M.L., Johnson N. (2016). Combination of the non-invasive tests for the diagnosis of endometriosis. Cochrane Database Syst. Rev..

